# Whole-genome sequencing identifies nosocomial transmission of extra-pulmonary *Mycobacterium tuberculosis*

**DOI:** 10.1093/qjmed/hcx115

**Published:** 2017-06-19

**Authors:** David A. Barr, Tom A. Yates

**Affiliations:** From the 1 Wellcome Trust Liverpool Glasgow Centre for Global Health Research, University of Liverpool, 70 Pembroke Place, Liverpool L69 3GF, UK; 2 Brownlee Centre for Infectious Disease, Gartnavel General Hospital, 1053 Great Western Road, Glasgow G12 0YN, UK; 3 Institute for Global Health, University College London, 3rd floor, Institute of Child Health, 30 Guilford Street, London WC1N 1EH, UK

We read this report with interest,^1^ but believe more clinical information is needed to confidently exclude airborne transmission of *Mycobacteriumtuberculosis*.

The pulmonary nodules identified by CT chest in the index patient (case 1) may have cavitated as part of an immune reconstitution syndrome. It would be useful to know if any respiratory symptoms developed during the admission or subsequently; the timing of the original CT in relation to the immune reconstitution syndrome; if any additional chest imaging occurred; and if respiratory specimens were obtained for mycobacterial culture at any stage.

Patients with HIV infection may be sputum culture positive despite a normal chest x-ray.^2^ Positron Emission Tomography Computed Tomography (PET-CT) scanning of asymptomatic individuals with ‘latent TB’ can reveal appearances similar to those seen in active disease.^3^^,^^4^ Serial imaging of pulmonary TB shows the disease to be highly dynamic.^5^ A significant proportion of ‘extra-pulmonary’ TB cases have concomitant pulmonary disease.^6^

There have been previous reports of *M. tuberculosis* transmission during aerosol generating procedures, including the irrigation of abscesses.^7^ Transmission via direct inoculation^8^ and ingestion are also described—here the latter is plausible, given the site of disease in case 2.

A better mechanistic understanding of which procedures are potentially aerosol generating might help guide practice. Ruling-out pulmonary disease is non-trivial, particularly in the context of HIV co-infection. Most transmission of *M. tuberculosis* is probably from individuals with pulmonary disease not yet on effective treatment (largely undiagnosed).^9^ This should be the focus of global efforts to prevent nosocomial transmission.

## Funding

DAB is funded through a Wellcome Trust PHD studentship grant ref 105165/Z/14/A.

*Conflict of interest*: None declared. 
